# Applications of Carbon Nanotubes in Bone Tissue Regeneration and Engineering: Superiority, Concerns, Current Advancements, and Prospects

**DOI:** 10.3390/nano9101501

**Published:** 2019-10-22

**Authors:** Baoqing Pei, Wei Wang, Nicholas Dunne, Xiaoming Li

**Affiliations:** 1 Key Laboratory for Biomechanics and Mechanobiology of Ministry of Education, School of Biological Science and Medical Engineering, Beihang University, Beijing 100083, China; pbq@buaa.edu.cn (B.P.); wangwei37@buaa.edu.cn (W.W.); 2 Beijing Advanced Innovation Center for Biomedical Engineering, Beihang University, Beijing 100083, China; 3 Centre for Medical Engineering Research, School of Mechanical and Manufacturing Engineering, Dublin City University, Stokes Building, Collins Avenue, Dublin 9, Ireland

**Keywords:** carbon nanotubes, bone regeneration, tissue engineering, scaffolds, drug system

## Abstract

With advances in bone tissue regeneration and engineering technology, various biomaterials as artificial bone substitutes have been widely developed and innovated for the treatment of bone defects or diseases. However, there are no available natural and synthetic biomaterials replicating the natural bone structure and properties under physiological conditions. The characteristic properties of carbon nanotubes (CNTs) make them an ideal candidate for developing innovative biomimetic materials in the bone biomedical field. Indeed, CNT-based materials and their composites possess the promising potential to revolutionize the design and integration of bone scaffolds or implants, as well as drug therapeutic systems. This review summarizes the unique physicochemical and biomedical properties of CNTs as structural biomaterials and reinforcing agents for bone repair as well as provides coverage of recent concerns and advancements in CNT-based materials and composites for bone tissue regeneration and engineering. Moreover, this review discusses the research progress in the design and development of novel CNT-based delivery systems in the field of bone tissue engineering.

## 1. Introduction

Millions of people around the world become victims of bone defects every year, due to the presence of arthralgia, osteoporosis, tumors, infection, congenital malformations, sports injuries, traffic accidents, natural disasters, etc. [1,2,3]. Although, unlike many soft tissues, bone has the ability to self-repair and regenerate when damaged, its self-regeneration ability is limited [3,4,5]. High-impact trauma or critical-sized bone defects may result in insufficient self-repair and require additional clinical treatment [6,7,8]. Approximately 4 million surgical procedures involve bone grafts or bone substitutes each year [9,10]. Currently, clinical treatment methods of bone repair consist of autografts, allografts, and xenografts [11]. The gold standard is autologous bone grafting with optimal capabilities of osteogenesis, osteoconductive, and no immunogenicity [12]. However, the disadvantages of autografts include a long operative time, insufficient donor sites, chronic pain, nerve injury, blood loss, and trauma complications [13,14]. Allografts are often considered as the next ideal candidate with osteoinductive and osteoconductive properties. However, allografts also significantly increase the risk of immunological rejection, infection, and transmissible diseases [15,16], and their mechanical properties are not adequate for load-bearing bone. To overcome these limitations of natural bone grafts, there is a huge demand for artificial bone substitutes that have similar physiological, chemical, and biological properties to natural bone. Therefore, bone tissue regeneration requires the development of safe and effective materials or composites, such as artificial bone substitutes, which has generated substantial interest in revolutionizing the clinical treatment of bone diseases and deformities.

Native bone is primarily composed of cells, fibrous protein, collagen, hydroxyapatite (HA, Ca_10_ (PO_4_)_6_(OH)_2_), and water, and has a nanocomposite structure that provides a skeleton for the human body [17]. In the aspect of bone tissue regeneration, considerable research efforts have been directed towards developing suitable materials or composites [18]. Currently, biomaterials used in bone tissue engineering mainly include natural polymer materials, synthetic polymer materials, and bioceramic materials. However, these three materials have different defects in the application of bone tissue repair. Natural polymer materials lack the ideal mechanical and electrical properties. Synthetic polymer materials have poor hydrophilic, cellular compatibility, and mechanical properties. Bioceramics, such as HA, have been widely used as ideal biomaterials in the repair of bone defects in plastic surgery or oral and maxillofacial surgery. However, HA alone cannot be used as bone substitutes for load-bearing bone or large-area bone defeats, due to the inherent shortcomings of poor plasticity, high brittleness, and low bending strength, which cannot meet the mechanical properties of bone tissue. Until now, there were no available synthetic bone substitutes, which have superior or even the same composition, structure, and properties of native bone under physiological conditions [19,20,21].

Advancements in nanotechnology have given rise to revolutionize the design and integration of innovative nanomaterial-based composites. Nanoscale characteristics have regulatory effects on cell hierarchy level and elicit favorable cellular behavior, including adhesion, proliferation, migration, differentiation, genetic expression, and signal transduction [22,23,24,25]. Among nanomaterials, CNTs have a reliable theoretical support as bone tissue engineering materials. Firstly, the good strength, elasticity and fatigue resistance of carbon nanotubes make them remarkable reinforcing materials in bone tissue engineering composite scaffolds. Carbon nanotubes can form a strong bond with the composite interface, which helps to effectively transfer load and improve the strength and toughness of matrix materials [26,27,28]. Secondly, the three-dimensional porous structure of CNTs had a high specific surface area, which is conducive to more protein adsorption and cell adhesion growth [29,30]. Thirdly, the interlinked nano-network structure and appropriate porosity of CNTs are conducive to the material exchange of extracellular matrix (ECM) in bone tissue. Their adjustable surface chemistry and high affinity for cell-binding proteins can be used to regulate cell morphology and promote stem cell differentiation into osteocytes, especially osteoblasts and neuronal lineage cells [31,32,33]. Next, their electrical conductivity is controllable by adjusting the diameter, length, and bending degree, which plays a role in regulating the physiological behavior of cells and serving as electron–cell biosensors [34,35,36,37]. Finally, their cylindrical shape and nanoscale dimensionality make them suitable for the delivery of aptamers, peptides, biomolecules, and various drugs [38,39,40,41].

This review paper describes unique properties and current concerns of CNTs as biomaterials for bone tissue regeneration and engineering. Recent advancements in CNT-based biomaterials and composites are reviewed as bone scaffolds or reinforcing agents. Moreover, the research progress in novel CNT-based delivery systems for the treatment of bone diseases is also discussed.

## 2. Advantages for Using CNT Composite Materials for Bone Tissue Regeneration and Engineering

**Figure 1:**
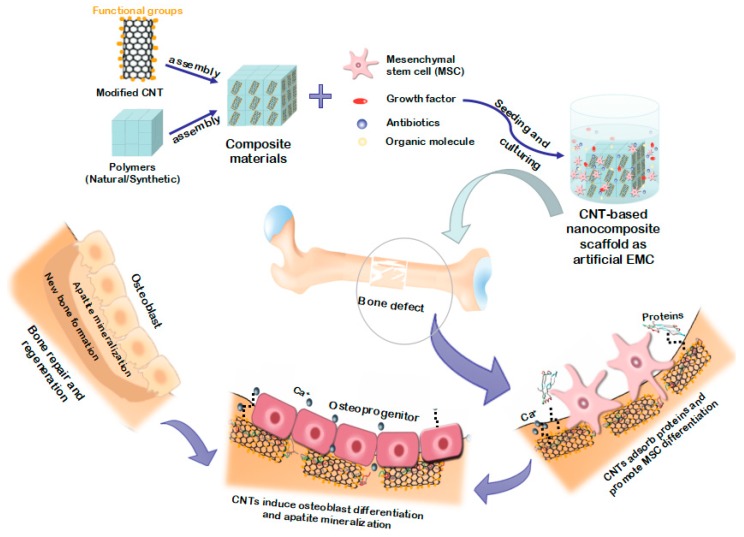
Schematic diagram describing the role of carbon nanotubes (CNTs) as scaffold composites in bone tissue engineering and regeneration.

Carbon nanotubes, as allotropes of carbon, consist of only carbon atom exclusively bonded to each other through sp^2^ bonds [42]. Carbon nanotubes can be seen as one graphene sheet rolled up into a hollow cylindrical nanostructure. Carbon nanotubes are commonly divided into two subtypes: single-walled carbon nanotubes (SWCNTs) formed from a single-layer of tubular graphene and multi-walled carbon nanotubes (MWCNTs) composed of multiple concentric tubular graphene layers. The SWCNTs usually present in a closely arranged bundle of hexagons, approximately 1 nm in diameter and up to a millimeter or more in length. The MWCNTs have a higher degree of structure similar to hollow graphite fiber, and a larger diameter than SWCNTs, ranging from 2 nm to 100 nm [43]. For bone tissue regeneration and engineering, CNTs in composites can be simply considered as biomimetic nanocomposites of collagen fibers on a cell hierarchy level. They can have a positive effect not only on stimulating cell adhesion through good interaction with cell-binding proteins [44,45,46,47], but also on regulating cell morphology and accelerating stem cell differentiation due to the fact of their preferential affinity for cell binding; thus, osteoblast differentiation and apatite mineralization were induced to promote new bone formation (Figure 1). Li et al. [48] demonstrated that MWCNTs can adsorb and concentrate more proteins (i.e., rhBMP-2), induce the expression of alkaline phosphatase (ALP) and the *cbfa1* and *COLIA1* genes, and then promote osteogenic differentiation of the human adipose-derived mesenchymal stem cells (MSCs) in vitro. Moreover, MWCNTs also induce ectopic bone formation in the dorsal musculature of mice in vivo, suggesting their ability to regulate the response of downstream stem cells without the incorporation of exogenous growth factors and other complex ligands. However, we must be concerned that single CNT materials are not suitable for cultivating regenerative bone tissue. Axial strength, toughness, and elastic modulus of pure CNT scaffolds are much higher than that of bone tissue, which is not well matched with human tissue. Only through composite application with other materials can CNTs give play to their unique mechanical, electrical, and surface properties, and then improve the overall physical and chemical features and bone conductivity of the composite materials.

### 2.1. Morphological Features

Carbon nanotubes have a seamless cylindrical morphology with nanoscale diameters, longer lengths, and large aspect ratios (100–100,000). The nanostructural unit of CNTs is a hexagonal honeycomb lattice of sp^2^-hybridized carbon atoms [49]. The sp^2^ forms strong in-plane C–C bonds without any dangling bonds, stronger than sp^1^ and sp^3^ bonds, which offer CNTs a high value of bond energy (614 kJ mol^−1^) [50]. The cylindrical shape and highly stable sp^2^ hybridized C–C bonds of CNTs are the vital ingredient for supporting their unique properties, including mechanical, chemical, thermal, and electrical properties [19]. At the nanoscale, bone can be simply identified as parallel type I collagen fibers arrays coated with plate-like carbonate apatite crystals. In the human physiological environment, the triple-helix tropocollagen macromolecules with a long rod-like shape generate the supramolecular collagen fibers though fibrillogenesis [51]. The rod-like shape and nanoscale of CNTs make it possible to use them as reinforcement materials to simulate collagen fibers in the ECM [52,53,54,55]. Currently, existing nanometer synthesis methods can prepare parallel or randomly orientated CNTs with a similar fibrillar hierarchy structure to ECM, and MWCNTs have been proven to reflect the band/gap structure of collagen as bone-like HA [56,57]. Furthermore, these tubular CNTs have an extremely large surface-area-to-volume ratio, making them suitable for supplying more sites for efficient adhesion of proteins, as well as providing higher drug loading capacity [58].

### 2.2. Mechanical Properties

High mechanical strength is a vital property of CNTs in the preparation of biomaterials in bone tissue engineering. Carbon nanotubes are not only flexible, but also have higher strength and lower density compared to conventional metals or ceramic. These ultralight CNTs with a density of 1.3–2.0 g/cm^3^ have a tensile strength of 11–52 GPa, bending strength of 14.2 ± 8 GPa [19], and Young’s modulus of 32–1470 GPa [59,60,61]. As one of the strongest materials known, they are at least 100 times as strong as steel and about three times as strong as bone. It has been proven that CNTs can significantly improve mechanical strength when a small number of CNTs are dispersed and mixed with polymers [62,63]. Although the dispersibility and interfacial adhesion of CNTs in the composites are poor, which increase local stresses and reduce failure energy, their incorporation is also capable of effectively increasing stiffness and toughness from several to ten times. Such a property provides a potential way to overcome the challenges that synthetic bone-like composites cannot have comparable mechanical properties to that of natural tissues and makes them an ultimate candidate as structural materials or reinforcements for polymer and ceramic matrices in the field of bone regeneration [64,65].

### 2.3. Chemical Properties

Carbon nanotubes have chemical stability and a strong adsorption capacity for most organic compounds [65,66]. The external surface of CNTs has a number of high-delocalized π-bonds that provide a prominent adsorption of proteins. However, CNTs are highly hydrophobic and hardly dissolve with any solvent. Their surfaces are usually modified or functionalized through introducing a large number of functional groups, such as hydroxyl, carboxyl, and alcohol groups [67,68,69]. There are two methods to functionalize the surface of CNTs: covalent and non-covalent [70,71,72,73]. The controllable functional chemical properties are of great significance, not only for its dispersion and biocompatibility in vivo environment but also for its conjugation with various biological molecules, such as growth factors, collagen, proteoglycans, glycoproteins and fibronectin, and bioactive materials, such as HA, calcium phosphate, and polymer materials. For better bone regeneration, studies have demonstrated that bone morphogenetic protein (BMP), amino acids, and type I collagen have shown good adsorption on surfaces of CNTs [74,75,76,77]. Functionalized CNT-based composites bonding with different proteins and other macromolecules can mimic the components of the ECM and further enhance cellular interactions and osteogenic potential. Indeed, bone remodeling sometimes requires the assistance of osteogenic drugs or proteins. Carbon nanotubes functionalized with assorted functional groups can act as a delivery vehicle for drugs, growth factors, proteins, and nucleic acids, helping controlling the behavior and differentiation of osteoblasts or osteoclasts in bone tissue [78,79,80,81,82].

### 2.4. Electrical and Magnetic Properties

Carbon nanotubes have unique electrical properties, ranging from metallic, semiconducting to superconducting conductivity and even different parts of the same CNT exhibit different electronic conductivity [83,84,85]. Their controllable electronic conductivity depends largely on their alignment, diameter, aspect ratio, and chirality. This unique performance enables CNTs to be used to study the effects of electrical stimulation on cell proliferation and activity, or as electronic biosensors for bioactive molecules like DNA, protein, and growth factors [45,86,87]. In bone tissue regeneration and repair, CNTs are capable of promoting the electrochemical and electron-conductive relativity of relevant biomolecules and proteins thus accelerating osteoblast proliferation and bone formation [88,89,90]. Carbon nanotubes can not only produce a high rate of cellular and osteogenic expression markers to form the plate-like crystals, but also maintain the electrical function of cell membrane and enhance the function of calcium ion channels [91]. Moreover, CNT-based composites are suggested as the best electroactive nanofibers, which can induce cells to align along the direction of electric charge in both randomly orientated and aligned fiber types, and show good antibacterial activity [92,93]. Thus, the electrical properties of CNT materials show the potential to regulate cell growth rate and induce tissue alignment during bone repair. Carbon nanotubes are inherently non-magnetic, but they can be made ferromagnetic by coating with nano-magnetic particles with different size, composition or structure on their surface or in tube cavity in a certain way [94,95]. Carbon nanotube-based magnetic composite nano-particles can effectively combine magnetic targeting technology with biological therapy, providing new theoretical basis for targeted therapy. More importantly, CNTs decorated with Fe_3_O_4_ showed enhanced biocompatibility characteristics that had a positive effect on cell behavior when exposed to an external magnetic field [94,95,96].

## 3. Concerns and Current Solutions of CNTs as Nanomaterials for Bone Tissue Regeneration and Engineering

### 3.1. Toxicity and Dispersity

Carbon nanotubes with good functional properties and structural properties hold great interest with respect to biomaterials for biomedical application in bone tissue regeneration and engineering. However, there are still many challenges in the clinical application of CNTs for the treatment of orthopedic diseases. At present, the toxicity and dispersity of CNTs are considered as the most concerning issues, greatly limiting their use as an ideal biological material.

The dispersity of CNTs is a major obstacle to the preparation of CNT–polymer nanocomposites, because of their hydrophobic nature [97,98,99,100,101]. The hydrophobicity of CNTs is due to the strong non-polar covalent bonds and hydrophobic surfaces of sp^2^ hybrid carbon atoms in CNTs [102,103,104]. These hydrophobic surface interactions, assisted by van der Waals forces and rod-shaped structures, tend to result in the congregation of CNTs in a bundle-like shape, which will greatly affect the mechanical, electrical, and thermal properties required for biomaterials in biomedical engineering applications. The main challenge in the preparation of CNTs is to prevent the formation of CNT aggregation and achieve their optimal dispersion into a polymer matrix [105,106]. Many studies have shown that functionalization is the most effective way to modify the surface of CNT under strong acidic conditions, including covalent and non-covalent [107,108,109]. Covalent functionalization is the formation of new chemical bonds with the surface of nanotube sidewalls through some chemical reaction, such as oxidation, hydrogenation, halogenation, halogenation, etc. Covalent functionalized CNTs can be combined with different polymers to prepare CNT–polymer composites with enhanced hydrocolloid stability and better dispersibility [110,111]. Non-covalent functionalized CNTs based on π–π interaction, hydrophobic interaction, and the van der Waals force can be used to adsorb and bond various functional groups, such as hydroxyl, phenyl, alkyl, alkinyl, ester, amino, acyl, and epoxy groups, the hydrophobic surface can interact with the hydrophobic portion of amphiphilic molecules, such as amphiphilic surfactants, polymers or biomolecules [112,113]. Polar solvents, such as dimethyl acetamide, ethanol, tetrahydrofuran, and dimethyl formamide, maintain stable dispersion of CNTs by inducing stronger repulsion among carbon nanoparticles. For example, the use of surfactants or other stabilizing agents are conductive to produce stable dispersions of CNTs by dispersing and exfoliating them.

In the field of tissue engineering, although CNTs have great application potential, its toxicological effects cannot be ignored. Nanoparticles can damage cell membranes by forming reactive oxygen species (ROS) and damage and cause cell death by inducing immune responses and chronic inflammation [114,115,116]. Existing studies have shown that the cytotoxicity and nanoscale activity of CNTs in living organisms are influenced by many factors, e.g., the size, surface area, agglomeration, processing method, and catalytic metal impurity [117,118]. Some groups have demonstrated that the length and diameter of CNTs have a significant effect on their toxicity [119]. In terms of length, the longer CNTs were more likely to cause inflammatory responses and granulomatous formation in cells and tissues than the shorter CNTs. In diameter, the cytotoxicity of SWCNTs was stronger than that of MWCNTs, and MWCNTs with a small diameter had greater toxicity than those with larger diameter [120]. Some studies also indicated that controlling the size of MWCNTs can produce different toxic effects on human health, because their length and diameter play an important role in determining surface modifications that may affect vector transfection efficiency [121,122,123,124,125]. Although the smaller size of CNTs improved cell and protein adhesion, the smaller diameter meant that it was more likely to affect cell membrane function and induce an immune response. On the other hand, CNTs longer than macrophages (20 μm) were not engulfed and degraded which then caused inflammation [107]. As the exact toxicity of CNTs is still unknown, their biosafety is a main concern for their application as potential nanobiomaterials in tissue engineering

### 3.2. Current Solutions for Biosafety

For bone tissue engineering, various researchers have found that CNT-based composites and substrates can purposefully interact with proteins, nucleic acids, and osteoblast-like human cells and contribute to better cell affinity, such as adhesion, proliferation, and osteogenic differentiation [126,127]. For this reason, the health risk of CNTs is considered as an inflammatory reaction on the surface of implants and scaffolds, resulting in their loosening and migrating from the desired implant location and the release of metal ions into surrounding tissues [63]. Once CNTs are released from scaffolds and implants, cross barriers reach blood circulation and, thus, disperse in the physiological environment, and they may become possible toxicological agents. In general, the potential cytotoxic effects of nanoparticles can be avoided by changing the method of synthesis and purification or properly modifying the surface of CNTs. Impurities, such as metallic contaminants and carbon particles, are also responsible for CNT toxicity. It is reported that these impurities introduced in the manufacturing process are toxic to cells and may cause gene-toxic reactions [128,129,130]. Until now, purification is still an important barrier to the preparation of very pure and well-characterized CNTs. Moreover, to develop the hydrophilicity of CNTs, hydrophilic groups (such as carboxyl and hydroxyl groups) need to be functionalized on the surface of CNTs through covalent or non-covalent binding. Based on the review of CNT toxic kinetics by Ali-Boucetta et al. [131,132], primitive and non-covalent functionalized CNTs may accumulate in the spleen and liver, while covalent functionalized CNTs may be excreted in the urine. Carbon nanotubes may have potential toxicity to human health in the process of production and application, which poses a great challenge to their clinical applications in bone regeneration; however, their toxicity has been conducted without conclusive solution on cell apoptosis and immune response. The toxicity of CNTs needs to be further characterized and accurately identified by substrates based on different cell lines and tested in vitro and in vivo. Considering these observations, many studies have proposed new strategies to reduce CNT toxicity based on the above factors, such as length diameter, metal impurity, and surface modification of CNTs [133,134,135,136]. Moreover, it is noted that preventing CNTs from entering the organism freely is also a prudent and effective method to reduce negative effects by limiting direct interaction with surrounding cells and tissues, such as wrapping CNT in hydrogels.

## 4. Advancements in CNT-Based Scaffolds or Implants for Bone Tissue Regeneration and Engineering

### 4.1. Synthesis Strategies for CNT-Based Scaffolds or Implants

The main methods for synthetization of CNTs are the arc discharge method, laser ablation method, and chemical vapor deposition (CVD). All of the above methods use carbon sources to form individual or groups of carbon atoms and then use arc discharge or thermal energy to recombine the atoms into carbon nanotubes [42,64,137]. The synthesis process of arc discharge and laser ablation techniques generally need to be conducted under a high temperature (>1700 °C), while CVD can obtain a relatively high purity of CNTs at lower temperature (<800 °C). In addition, other non-standard methods are also used, such as solid pyrolysis, hydrothermal treatment, and laser sputtering [138,139].

There are a variety of processing techniques for preparing CNT-based composite scaffolds. Different manufacturing processes are usually chosen according to the composition of the composites [44,51,118]. The fabricating methods of CNT-reinforced ceramic scaffolds generally include solution casting, hot pressing process, laser sintering, microwave sintering, and in situ precipitation. On the other hand, lyophilization, sol-gel method, phase separation method, CVD, solution mixing method, 3D printing, and electrospinning technology are commonly used to fabricate CNT-based polymer scaffolds. Due to the diversity of materials, each scaffold manufacturing technique has its relative advantages and different application areas [18,83]. It is very important to choose a scaffold manufacturing technique that is suitable for the properties of CNT-based composite materials.

### 4.2. CNTs with Calcium Phosphate Materials

Bone is hierarchy composed of 30% organic matter and 70% inorganic matter, while calcium phosphate, mostly HA, accounts for 95% of inorganic components. Bioactive calcium phosphate-based materials have been profusely studied as biomaterials for bone tissue regeneration and repair owing to their similar compositions and structures with a natural mineral phase of bone, good osteoconduction, and bone-formed ability [140,141,142].

The representative HA, TCP, and calcium phosphate cements (CPCs) have been profusely studied as biomaterials for bone tissue regeneration and repair. Nevertheless, the inherent defects of these biomaterials are brittleness, low flexural/tensile/fracture strength, as well as shaping difficulty [143,144,145,146]. Taking advantages of their extraordinary mechanical strength, CNTs have been reported to be an ideal candidate for enhancing the mechanical properties and biological performances of calcium phosphate-based materials.

**Figure 2:**
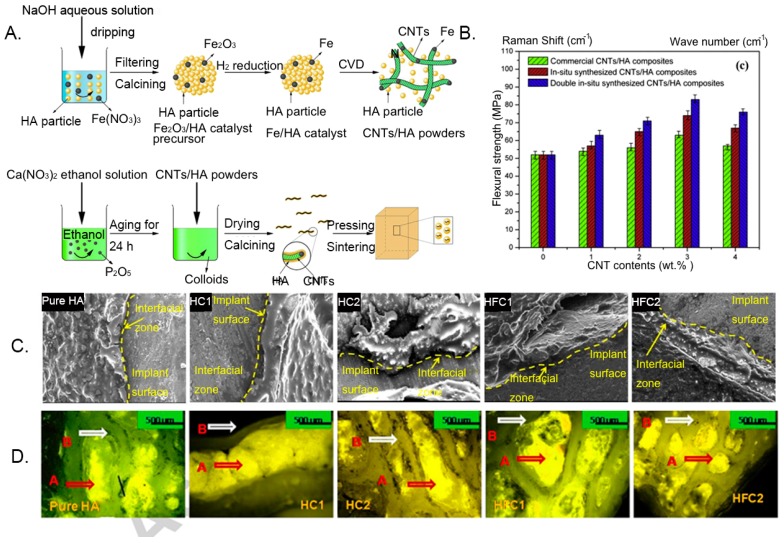
Optimized bioavailability of a carbon nanotube–hydroxyapatite (CNT-HA) composite in bone engineering. (**A**) Schematic diagram of the preparation process of the CNT–HA composite. (**B**) Flexural strength of HA composites with different contents of CNTs. (**C**) SEM micrographs of the host–implant interface 120 days after implantation. (HA: hydroxyapatite; HC1:HA + 1% WCNT; HC2:HAC + 2% MWCNT; HFC1:HA + 1% functionalized-MWCNT, and HFC2:HA + 2% functionalized-MWCNT). (**D**) Fluorochrome labeling images at 120 days after implantation showing new bone (golden yellow) and old bone (deep sea green). Reproduced with permission from [147,148]. Elsevier, 2016.

Hydroxyapatite, with good biocompatibility, is good at simulating the natural function of bone; therefore, it is widely used in artificial bone-like scaffolds. However, its tensile strength and fracture toughness are weak in bearing substantial loads, such as bone, due to their highly brittle nature. The mechanical properties of CNTs can be used to optimize the bioavailability of HA in bone engineering. For instance, Li et al. [147] first prepared CNT-reinforced HA composites successfully through a double in situ synthesis of the chemical vapor deposition and sol-gel methods (Figure 2A). The thickness, morphology, and structure of HA layers can be controlled by adjusting the amount of the CNT–HA composite during modification. The flexural strength of CNT–HA composites was increased to 83 MPa, which was approximately 1.6 times higher than that of pure HA (Figure 2B), due to the homogeneous dispersion of CNTs and strong interfacial bonding between the CNTs and HA matrix. These CNT–HA composites also presented good biocompatibility and significantly promoted the proliferation of fibroblasts and osteoblast in vitro. In the other study, Mukherjee et al. [148] also indicated the flexural strength and impact strength of the composites were significantly improved, and fracture toughness of the composites was increased to 1.9 MPa.m1/2, which is close to that of human cortical bone (2–4 MPa.m1/2). The results on the defect site of the rabbit model in vivo showed that bone integration was enhanced at the host–graft interface, and the reticular soft callus grew from the host surface to the implant surface (Figure 2C,D), ensuring a good host–bone interaction. Combined with good biocompatibility and mechanical properties, the various CNT–HA composites were not only designed as bone scaffolds, but were also developed as a coating, hybrid powder or nanocomplex for the use of three-component systems in the bone biomedical application. MWCNT–HA composites were synthesized to coat the surface of pure titanium in order to improve bioactivity. The results found that HA crystals were more likely to form on the surface of titanium coated with MWCNT–HA particles, indicating the surface of CNTs was suitable for promoting apatite mineralization and accelerating new bone formation and bonding with coated implants [149]. Moreover, the MWCNTs–HA was synthesized with collagen to fabricate the bionic scaffolds with nanostructure, constituents, and mechanical features of native bone [150]. The CNT–collagen–HA was about 10 times stronger than collagen–HA and promoted proliferation and diffusion of bone marrow mesenchymal stem, as well as mRNA and protein expressions of bone salivary protein and osteocalcin. Carbon nanotubes in the HA-based composites are generally considered to replace collagen fibers as structural stiffeners for bone tissue scaffolds. Hydroxyapatite composites with CNTs offer an opportunity to expand the spectrum of properties without affecting biological activity, which is crucial for biomedical applications.

Tricalcium phosphate has a similar chemical composition and structural and elastic moduli to HA, known as another important calcium phosphate-based biomaterial. Beta-tricalcium phosphate (β-TCP) not only possesses good biocompatibility and osteoconductivity, but also shows in vivo resorbability during the bone growth, making it an ideal candidate as a biomaterial for bone tissue engineering [151,152,153]. Mirjalili et al. [154] successfully synthesized a β-TCP–CNT nanocomposite by solution precipitation method. The β-TCP nanocomposite with 1 wt% CNT provided the best microstructure without agglomeration. Hydroxyapatite was deposited on the surface of the nanocomposite after immersion in simulated body fluid after 7 and 14 days, indicating the in vitro bioactivity of the β-TCP–CNT nanocomposite, because negatively charged groups of this nanocomposite were able to induce apatite formation by the adsorption of calcium (Ca^2+^) ion and subsequent complexation [155]. Taken together, incorporating CNTs into a β-TCP composite can provide a nucleating site for the apatite formation and enhance HA formation after implantation. Although this β-TCP–CNT nanocomposite is a candidate as bone implant materials, the actual behavior of cells on the surface of this nanocomposite must be further confirmed in the body.

Calcium phosphate cements is the hydration reaction of a mixture of one or more calcium phosphate powders with a mixed-liquid phase. Some CPC formulations are now commercially available; however, due to the fact of their limited compressive strength, they are restricted primarily to stress-free applications, such as dental fillings, maxillofacial prosthesis, and cranial repair [156,157]. Chew et al. [158] developed high compressive strength CPCs (β-TCP and di-calcium phosphate anhydrous) by incorporating with MWCNTs and bovine serum albumin (BSA) for bone repair. A functionalized MWCNT–CPC composite exhibited the highest compressive strengths (16.3 MPa) which increased by more than 10 times compared to pure cement (≈1 MPa), within the range of values for trabecular bone (2–12 MPa). In addition, through the effective attraction of both Ca^2+^ and PO^3−^, the presence of MWCNTs promoted the nucleation, growth, and formation of HA crystals. The CPC–MWCNT composite demonstrated a high injectability (97%) thus being suitable for bone repair applications as in situ hardening cement.

### 4.3. CNTs with Natural Biopolymers

Natural biopolymers, such as chitosan, collagen, fibrin, and hyaluronic acid have been proven to be promising biomaterial for bone scaffolds or implants due to the fact or their unique biocompatibility and osteoconductivity. However, due to the fact of its uncontrolled degradation rate, their low mechanical stability is an uncertainty when simulating the mechanical and biological characteristics of natural bone tissue matrix. Although CNTs are poor in hydrophobicity and immiscible with water, the functionalized CNTs modified with hydrophilic groups, such as –OH and –COOH, exhibit dissolubility in water. The addition of CNTs in the polymer matrix is expected to improve the beneficial properties of materials by forming strong hydrogen bonds. Recently, some progress has been made in the application of CNT–biopolymer nanocomposites in bone tissue engineering.

**Figure 3:**
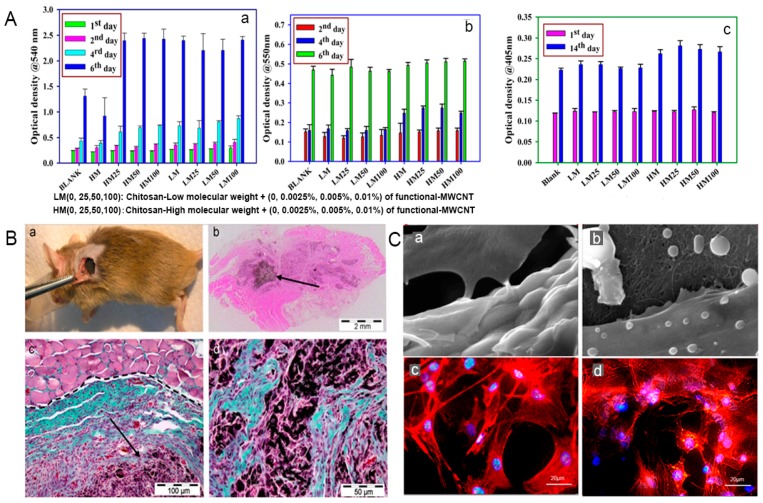
Carbon nanotube-reinforced chitosan (CNT-CS) biomaterials with in vivo and in vitro bioactivity and mechanical strength. (**A**) MTT assay of the viability of an MG-63 osteoblast-like cell; (**b**) protein estimation; and (**c**) Alkaline phosphatase (ALP) activity of an osteoblast-like MG-63 cell on different CS–MWCNT composite scaffolds as a function of time. (**B**) The surgical implantation of (**a**) rhBMP-2 adsorbed MWCNT–CS scaffolds into the subcutaneous muscular pocket of a mouse; (**b**) optical microscope micrograph of regenerated bone tissue; (**c**,**d**) optical micrograph in detail of regenerated bone tissue (blue–green), the remaining scaffold (black), and plenty of fibroblasts (purple colored) after major disassembly of the MWCNT/CS scaffold, surrounded by muscle tissue (pink). (**C**) MC3T3-E1 cells spreading on interconnected porous HA ceramic (**a**,**c**) and 3D-porous CNT (**b**,**d**) scaffolds (actin filaments: red; nucleus: blue). Reproduced with permission from [159,163,164]. Elsevier, 2012, 2008; MDPI: Open Access, 2017.

Chitosan (CS), a linear polysaccharide derived from chitin through deacetylation, has played a major role as a promising biomaterial with broad application prospects, due to the fact of its good biocompatibility, biodegradation, and antibacterial activity [159]. Chitosan can be easily converted into various geometries and forms for cell ingrowth and osteoconduction, and it has a cationic nature and hydrophilic surface which is beneficial for attracting various negatively charged proteoglycans and promoting the mineralization of the bone matrix after implantation [160,161]. These characteristics make CS a unique attraction as a new biomaterial for bone tissue engineering. Nevertheless, the most challenging part is its low flexibility and mechanical properties, which cannot support weight-bearing bone grafting [100]. Carbon nanotubes are an important reinforcement phase to improve the properties of CS, when they are strongly bonded and uniformly dispersed in the CS matrix. The functionalization of CS and CNTs can effectively enhance the interactions between inorganic and organic phase, and a higher energy is needed to overcome the molecular bonding energy thus significantly improving the mechanical properties of this substrate. Ashkan et al. [62] reported that when 1 wt.% of MWCNTs was homogeneously dispersed throughout the CS matrix, the elastic modulus and tensile strength of the composite were greatly enhanced by 47% and 33%, respectively. Similarly, Wang et al. [157] also indicated that the CS matrix blended with only 0.8 wt.% MWCNTs exhibited significant improvements in the tensile modulus and strength, ranging from 1.08 GPa to 2.15 GPa and from 37.7 MPa to 74.3 MPa, respectively. In another study, possible utilization of CS–CNT-based nanocomposite has been reported to be an effective method to improve the beneficial mechanical properties of HA in bone tissue engineering. Chen et al. [162] found that when MWCNT–CS weight ratios ranged from 0 wt.% to 5 wt.%, the compressive strength and elastic modulus of CS–MWCNT–HA composites increased rapidly from 33.2 to 105.5 MPa and from 509.9 to 1089.1 MPa, respectively. Although these synthetic composites did not obtain the strength matched with natural bone, the properties of CNT–CS composites still had great room for improvement with the optimization of dispersion and interface interaction between CNTs and CS. From a biological point of view, functionalized CS–CNT composites not only exhibit non-toxic effects but also promote stem cell differentiation into bone-forming cells. The in vitro study demonstrated the good biocompatibility of CS–CNT composites. The study also reported that the osteoblasts cultured on the CS–CNT composite adhered strongly to the surface, and the morphology was normal after 3 and 7 days without cytotoxicity [62]. The cell proliferation rate of MG-63 cells (human osteosarcoma cell line) on the CS–MWCNT scaffold was twice that of pure a CS scaffold, and the composite scaffolds also showed higher protein content, ALP, and mineralization than those of CS scaffolds (Figure 3A) [159]. For the in vivo response of MWCNTs, the CS–MWCNT scaffold incorporated with recombinant human bone morphogenetic protein-2 (rhBMP-2) showed the absence of chronic inflammation throughout the period, and bone tissue regeneration was observed at 3 weeks after implantation (Figure 3B) [163]. Moreover, some tricomponent composites showed high biological activity as bone scaffolds. For instance, preosteoblast MC3T3-E1 cells possessed good attachment and adhesion on the surface of the CS–MWCNT–HA composites, and the cell proliferation and growth morphology of MC3T3-E1 on the CS–MWCNT–HA composites were significantly better than that on the CS–HA composites (Figure 3C) [164,165]. In an in vivo experiment of CS–CNT membranes implanted into cranial defects in rats, this composite did not cause chronic inflammatory over a period of 5 weeks [94,166]. Another tricomponent composite scaffold, silver sulfadiazine–MWCNT incorporated into CS nanofibers were used as a coating to improve antibacterial properties of Mg–Zn–Ca alloy implants for bone treatment [167]. These CS nanofibers with 0.5 wt.% silver sulfadiazine–MWCNTs had exceptional antibacterial performance toward *Staphylococcus aureus* and *Escherichia coli* and resulted in better cellular compatibility proliferation thus reducing the incidence of bone infections. These CS–CNT bicomponent or tricomponent composites can improve the mechanical properties and bioactivity and promote the differentiation and growth of osteoblasts in bone regeneration. However, further systematic research is needed to study and design CS–CNT-based biomaterials with in vitro and in vivo bioactivity and mechanical properties for achieving a biocompatible internal environment in human physiological conditions.

**Figure 4:**
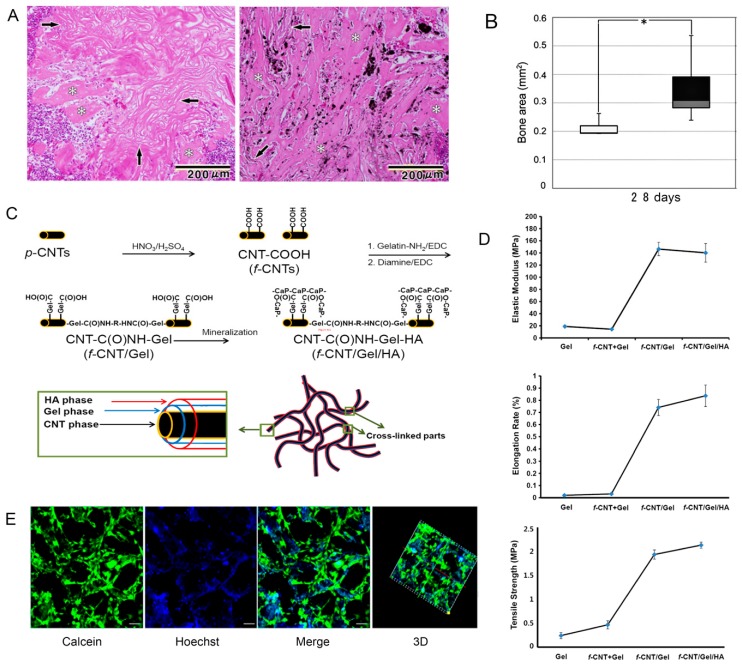
Carbon nanotube (CNT)-based collagen and gelatin natural polymer matrix for bone formation. (**A**) Histology of bone formation (white asterisk) at 28 days after bone marrow implantation. The uncoated sponge on the left is surrounded by a small amount of newly formed bone; the MWCNT-coated sponge on the right is surrounded by a large amount of newly formed bone. (**B**) The amounts of new bone formed around the MWCNT-coated scaffold are significantly higher than that around uncoated scaffolds at 28 after implantation (* *p* < 0.05). (**C**) Schematic demonstration of the preparation of functionalized CNT–Gelatin (Gel)–hydroxyapatite hybridized nanofiber assembly, which is comparable to collagen fibers in natural bone structure. The preparation schematic diagram of functionalized CNT–Gel–hydroxyapatite (HA) composite, which is similar to natural bone structure. (**D**) Elastic modulus, tensile strength and elongation rate of Gel: gelatin, f-CNT + Gel:functionalized-CNT + gelatin, which were prepared only by physical mixing, f-CNT/Gel:functionalized-CNT+gelatin, and f-CNT/Gel/HA:functionalized-CNT + gelatin + hydroxyapatite membrane samples. (**E**) The distribution and viability of MC3T3-E1 cells on Chitosan–gelatin–HA-0.6% MWCNT scaffolds (incubation for 2 days, scale: 50 μm). Calcein (green) staining shows the spread of live cells, Hoechst (blue) staining shows the cell nucleus. Reproduced with permission from [175,176,177]. Elsevier, 2011, 2014; MDPI: Open Access, 2019.

Collagen are the predominant organic phase of bone which are responsible for the resilient nature of bone, such as the toughness and viscoelasticity. Collagen has been clinically applied as a promising biomaterial to regenerate bone tissue due to the fact of its biodegradation, low antigenicity, and cytocompatibility [168,169,170]. Pure collagen are relatively soft (elastic modulus: 14.6 ± 2.8 kPa) and cannot be directly used as bone replacement materials [171]. The combination with CNTs is expected to optimize properties of collagen materials thus making them suitable for use as biomimetic composite scaffolds in bone tissue engineering. Tan et al. [172] indicated that the incorporation of covalently functionalized CNT in collagen-based constructs was an effective means of improving mechanical stability by restructuring collagen fibrils and forming robust thicker fibrils. Because covalently functionalized CNTs are capable of strongly binding to collagen molecules, they can induce the formation of larger fibril bundles. Carbon nanotubes in the collagen matrix have been proven to promote bone differentiation and bone regeneration mineralization [173,174]. For instance, Hirata et al. [175] demonstrated that an MWCNT-coated 3D collagen scaffold accelerated the early differentiation of osteoblasts compared with an uncoated collagen scaffold and induced new bone formation in the pores after 28 days of implantation in the femur (Figure 4A,B). Moreover, further investigation of the MWCNT–collagen-based materials also indicated their good in vivo osteogenic-inducing capacity as biomimetic scaffolds. When the 0.5% MWCNT–collagen–HA scaffold was implanted into critical-sized calvarial defect in a rat, no chronic inflammation and inflammatory cell infiltration were observed during the whole repair process. At 12 weeks post-implantation, the calvarial defect was covered with newly formed bone and dense connective tissue, and new bone was still directly attached to the MWCNTs, indicating that the MWCNTs demonstrated a nucleating function for HA precipitation [150]. Therefore, it is necessary to develop CNT-reinforced collagen and calcium phosphate nanocomposites, which have the advantages of good mechanical strength, high porosity, high hydrophilicity, good biocompatibility, etc. and are closer to natural bone.

Gelatin, a degraded derivative of structural collagen, has better cost effectiveness and common availability than collagen. At the same time, it has the advantages of resilience, biocompatibility, biodegradability, and lower antigenicity similar to collagen, making it an inexpensive protein polymer for bone tissue engineering applications. Although there have been few reports of gelatin composites directly synthesized with CNTs, CNTs have been used as enhancers for HA– or CS–gelatin nanocomposites in the application of artificial bone grafting. An artificial bone graft, consisting of a CNT core and gelatin–HA shells, was indicated as having a similar structure and composition to natural bone (Figure 4C). Compared to pure gelatin, the elastic modulus, tensile strength, and elongation rate of the new multilayered CNT–gelatin–HA core–shell material were significantly increased by 9–10, 4.6–8.8, and 28–42 times, respectively (Figure 4D), and this composite also had a higher degree of biocompatibility and cell viability [176]. As evidenced by other studies, the addition of MWCNTs to the CS–gelatin–HA composite scaffolds effectively improved the mechanical strength of the scaffolds, with the maximum strength of 6.58 MPa, which is approximately 3.4 times of the elastic modulus of CS–gelatin–HA scaffolds. In the CS–gelatin–HA-0.6% MWCNT scaffold, MC3T3-E1 maintained good morphology and possessed cell adhesion, proliferation, and osteogenesis differentiation in vitro, providing a good foundation for the formation of new bone (Figure 4E) [177]. Therefore, gelatin is a promising scaffold material with potential application in the field of bone tissue regeneration.

In addition to the above biomaterials, CNTs are also used to combine with other natural polymers with the aim of enhancing their biological and mechanical characteristics [34,51,52,178]. Compared with bacterial cellulose matrix, functionalized MWCNTs mixed with natural bacterial cellulose improved the mechanical properties of bone scaffolds and supported the active adhesion and proliferation of osteoblasts at a higher level [179]. Moreover, silk fibroin films prepared by incorporating MWCNTs also have good biocompatibility, supporting bone marrow stem cell (BMSC) adhesion and growth for more than 7 days [180]. Thus, the findings from these studies highlight the design and development of novel and safe CNT-based natural biopolymer scaffolds and implants in bone tissue regeneration and engineering.

### 4.4. CNTs with Synthetic Biopolymers

Broad synthetic polymeric materials have been extensively studied as scaffolds and implants for tissue engineering. However, the use of these synthetic biopolymers materials is limited in bone tissue repair, because of their specific disadvantages, such as poor mechanical strength, low osteoinductive capacities, and difficult structural designs. Carbon nanotube materials have been successfully used as reinforcement materials to introduce their significant physical and chemical properties into synthetic biopolymers, expecting to obtain ideal composite scaffolds for bone regeneration.

**Figure 5:**
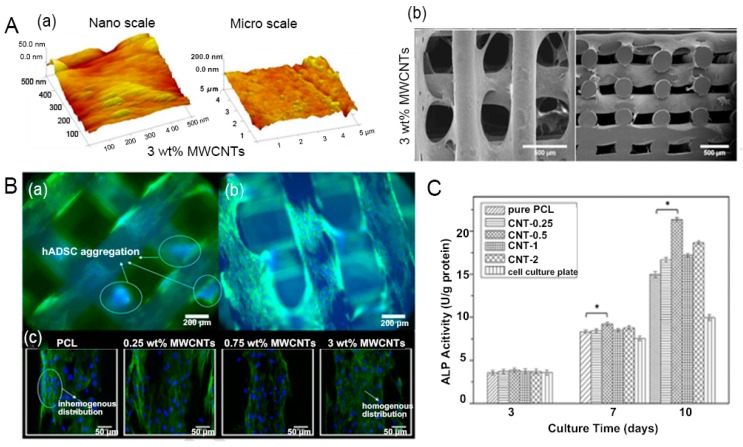
Carbon nanotube- polycaprolactone (PCL–CNT) scaffolds used for bone regeneration (**A**): (**a**) AFM images of the surface topography of the printed PCL–MWCNT scaffolds; (**b**) SEM images of cell morphology at day 14 on the PCL-MWCNT scaffold. (**B**) Fluorescence microscopy images of (**a**) PCL and (**b**) PCL-3 wt.% MWCNTs; and (**c**) confocal images of cell morphology on surfaces of all PCL–MWCNT scaffolds at 14 day. (**C**) ALP activity of bone marrow stem cells (BMSCs) cultured on different scaffolds at 3, 7, and 10 days, * *p* < 0.05. Reproduced with permission from [187,189]. Elsevier, 2019, 2012.

Polycaprolactone (PCL), a semi crystalline polymer, is widely used as a tissue-engineered scaffold material for bone tissue, because of its good biocompatibility, biodegradability, drug permeability, and ease of processing [181,182,183,184]. However, PCL shows high hydrophobicity, poor cell affinity, low bioactivity, and insufficient mechanical properties for load-bearing substances. The preparation of the composite with other materials, such as CNTs, is an effective strategy to overcome these limitations [185,186]. For instance, the PCL–MWCNT scaffold prepared by a screw-assisted extrusion-based additive manufacturing presented evenly distributed regular pores [187]. The incorporation of MWCNTs into the PCL matrix yielded significantly higher compressive modulus of 88 MPa, close to the modulus of cancellous bone (100–5000 MPa) [188] and changed the surface roughness and topography of the scaffolds (Figure 5A). This composite scaffold, with pore sizes ranging from 366–397 μm, supported early-stage human adipose-derived MSCs’ attachment and proliferation and increased protein adsorption (Figure 5B). In a similar study, 3D-porous scaffolds of MWCNT–PCL nanocomposites were fabricated by the solution mixing and evaporation technique [189]. The addition of 0.5 wt.% MWCNTs showed the best improvement in the tensile and compressive strength of the composite, which facilitated proliferation and differentiation of rat BMSCs into the osteogenic lineage, with high levels of bone marker ALP (Figure 5C). In another in vitro and in vivo study, PCL–HA slurry containing uniformly dispersed ionically modified 0.2 wt.% CNTs increased in compressive strength and elastic modulus by 1.5 times and 2.5 times, respectively, compared to the PCL–HA scaffold [190]. This slurry also showed favorable in vitro bioactivity, which induced substantial apatite mineralization in the simulated body fluid and accelerated MC3T3-E1 cell proliferation in vitro. When this scaffold was implanted into a rat subcutaneous tissue for 4 weeks, soft fibrous tissues with new blood vessels were present in the pore channels of the scaffold and there were no obvious signs of inflammation. These PCL–HA–CNT scaffold developed promising new candidate substance, supporting cellular growth, angiogenesis, and tissue development for bone regeneration.

**Figure 6:**
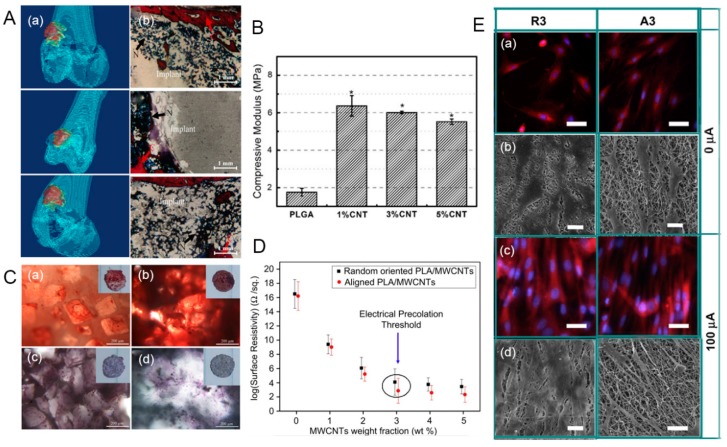
The application of carbon nanotube (CNT)-based polymethyl methacrylate (PMMA) and poly(lactide-co-glycolide) (PLGA) biomaterials for bone regeneration and fracture healing. (**A**) 3D reconstruction of CT images (**a**) and van Gieson-stained images of PMMA–MWCNT bone cement specimens at 12 weeks after implantation (**b**) (collagen fibers: red; nucleus of osteoblast: brown-black). (**B**) Compressive modulus of PLGA and CNT–PLGA scaffolds (* *p* < 0.05). (**C**) Alizarin red S staining (**a**,**b**) and alkaline phosphatase (ALP) staining (**c**,**d**) of MC3T3-E1 osteoblasts on PLGA (**a**,**c**) and 1% CNT–PLGA scaffolds (**b**,**d**) at day 21. (**D**) Impact of level of MWCNT loading on the surface resistance of electrospun polylactic acid (PLA)–MWCNT nanofiber meshes. (**E**) Fluorescence microscope images (**a**,**c**) and selected scanning electron microscope images (**b**,**d**) of osteoblasts cultured on R3 (randomly oriented PLA fibers with a 3 wt.% ratio of MWCNTs) and A3 (aligned oriented PLA fibers with a 3 wt.% weight ratio of MWCNTs) for 5 and 7 days, respectively. Reproduced with permission from [192,195,199]. Elsevier, 2019, 2013 and 2013.

Polymethyl methacrylate (PMMA) bone cement, as a grouting agent, has been used in orthopedic surgery for decades. The PMMA bone cement forms a critical interface by filling the free space between the implant and the bone and plays a load-transferring role in vivo. Thus, it bears a repetitive loading pattern generated at the interface during daily activities, which determines the long-term survival of the implant prosthesis. However, PMMA bone cement is prone to fracture due to the tensile stress and accumulation of fatigue damage. Many research groups have highlighted the potential of CNT in PMMA bone cement to improve fatigue performance and encourage interfacial cell growth. Ormsby et al. [191] found that the incorporation of MWCNTs significantly augmented the fatigue properties of PMMA bone cements. Due to the high dispersion of MWCNT in the cements, preventing or delaying the expansion of cracks in cements through the bridging effect and MWCNT pull-out, the 0.1 wt.% MWCNT–COOH provided the greatest improvement in the fatigue life of PMMA bone cements. In addition, MG-63 osteoblasts successfully adhered and proliferated on the surface of the MWCNT–PMMA bone cements within 7 days, indicating their necessary biocompatibility to allow cell adhesion and growth. In another study, Wang et al. [192] also demonstrated that the incorporation of MWCNTs improved cytocompatibility and osseointegration of PMMA bone cements. The results of in vitro rat BMSC culture on the composite showed that MWCNT–PMMA bone cements not only improved cell adhesion and proliferation, but also increased the osteocalcin gene and protein expression which promoted the osteogenic differentiation. Using an in vivo New Zealand rabbit bone defect model study, 1.0 wt.% MWCNTs increased the bone ingrowth ratio by 42.2% and induced massive osteoblast congregation and new bone formation surrounding the bone cements at 12 weeks after implantation (Figure 6A). Moreover, Ormsby et al. [193] also found that chemically functionalized MWCNTs had also been reported to overcome the problem of the heat generated during the solidification and polymerization of PMMA bone cement via changing its polymerization kinetics, reducing the polymerization rate and exothermic reaction. Hence, MWCNT-reinforced PMMA bone cements have broad application prospects in orthopedic applications.

Poly(lactide-co-glycolide) (PLGA) is one of the most popular synthetic polymers that have been widely used as materials, although they are not strong enough to withstand mechanical loads as bone substitute materials. The addition of CNT to PLGA can overcome challenges regarding mechanical properties. Mikael et al. [194] indicated that the 3D PLGA scaffold containing only 3% water-dispersible MWCNTs had a significant increase in compressive strength and modulus (35 MPa, 510.99 MPa) compared to pure PLGA scaffolds (19 and 166.38 MPa), and showed good cell viability, proliferation, and mineralization. Cheng et al. [195] used the solvent casting/particle leaching technology to prepare a CNT–PLGA scaffold for bone repair. The homogenous dispersion of CNTs in the PLGA substrate enhanced the mechanical strength and surface roughness, thereby increasing the attachment and proliferation of MC3T3-E1 osteoblasts. This CNT–PLGA scaffold achieved a 3.4 fold increase in compression modulus and a higher degree of adhesion and proliferation rate of MC3T3-E1 osteoblast-like cells, leading to in vitro bone mineralization (Figure 6B,C). In another similar example, the carboxyl-functionalized MWCNT-modified PLGA nanocomposite yielded an increase in the tensile strength of 11.3 ± 1.3 MPa and in the elastic modulus of 375 ± 20 MPa in comparison to 4.1 ± 0.5 MPa and 67 ± 10 MPa, respectively, for the PLGA matrix. The MSCs cultured on the scaffold also exhibited better adhesion and viability and significantly increased the production levels of ALP over 21 days of culture [196]. Taken together, these studies suggest potential applications for the development of 3D CNT–PLGA scaffolds on bone tissue engineering.

Polylactic acid (PLA) or poly-L-lactic acid (PLLA) are a biodegradable aliphatic polyester extracted from abundant and renewable resources. Among biodegradable polymers used for bone engineering, PLA has good modeling performance, controllable degradation, and good histocompatibility, which are considered as useful characteristics for bone regeneration. Nevertheless, the inherent brittleness and poor thermal stability of PLA make it unable to withstand heavy loads or stimulate cell proliferation. Incorporating CNT agents to the PLA matrix can optimize its mechanical properties and surface functionalization, thus promoting cell viability. For instance, the PLA–CNT–COOH composite prepared by the melt blending technique yielded an increase in the tensile strength, elongation at break, and impact strength simultaneously, and obtained a higher thermal stability than pure PLA [197]. Similarly, MWCNTs combined with a pyrene-end-functionalized PLLA dispersing agent were well dispersed in the PLLA matrix [198]. The Young’s modulus of the MWCNT–PLLA scaffold was significantly increased up to 2625 ± 90 MPa; this scaffold also supported the adhesion and proliferation of BMSCs in vitro and retained cell potential to undergo osteogenic differentiation. In another interesting study, the electrically conductive PLA nanofibers embedded with MWCNTs were fabricated to study the synergistic effect of electrical stimulation and topographic cues on osteoblasts outgrowth (Figure 6D) [199]. Electrical stimulation significantly promoted elongation of osteoblasts and guided the outgrowth of osteoblasts along the nanofiber direction (Figure 6E). Therefore, this interesting study suggested that electrical stimulation imparted on a conductive PLA–MWCNT substrate has a great potential application in coupling bone regeneration and fracture healing.

Many synthetic polymers, such as polyvinyl alcohol (PVA), poly glycolic acid (PGA), poly(etheretherketone) (PEEK), polyanhydrides poly(propylene fumarate) (PPF), polypropylene, and polyurethane, have been combined with CNT to enhance and optimize their physicochemical features and structural form through many different techniques, including electrophoretic deposition, freeze drying, laser sintering, particulate leaching, solvent casting, and gas foaming [200,201,202,203,204,205,206]. The addition of CNTs resulted in a significant increase in cell adhesion, osteoblast growth, ALP, and bio-mineralization. These synthetic nanocomposites have potential applications in the development of in vivo characterization and fabrication of 3D scaffolds for bone tissue regeneration and engineering. In general, CNTs as reinforcements in composites can not only support cell adhesion, growth, and proliferation, such as for bone marrow stem cells, human osteoblasts, fibroblasts, and periodontal ligament stem cells, but can also induce osteoblast differentiation and apatite mineralization and promote new bone formation. Many researchers have spared no effort to conduct a great deal of research and design new strategies for applying nanomaterials as scaffolds, coating films or additives to improve the performance of certain substrates of bone tissue, as summarized in Table 1.

## 5. Advancements of CNT Composite as Nanocarriers for Bone Tissue Regeneration and Engineering

**Figure 7:**
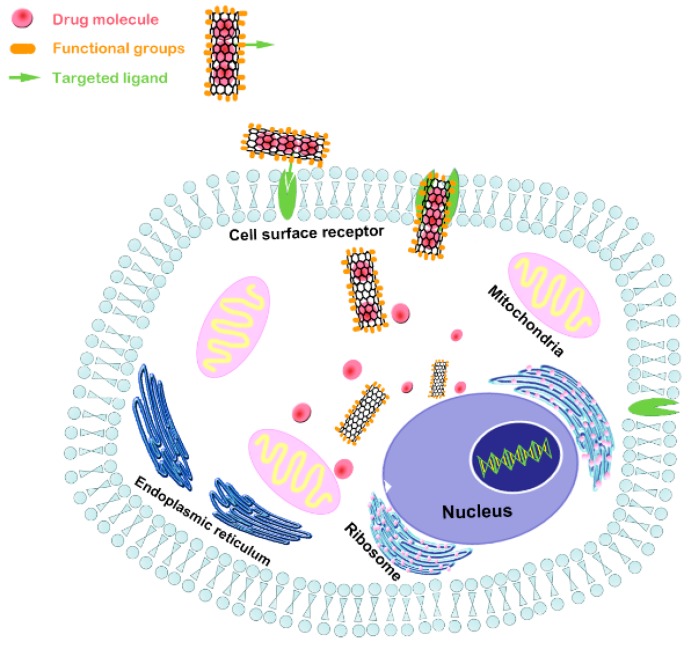
A CNT-based drug delivery system. Due to the poor cell penetration of many small molecules and an increasing number of large molecules, CNTs functionalized with a targeted ligand were able to penetrate cell membranes through an ion channel and transport-specific drug molecules into targeted cells.

Carbon nanotube, as nanocarriers of drugs, genes, proteins, and other delivery systems, have exhibited good efficacy in the treatment of many diseases. Due to the fact of their unique properties, such as CNT-based delivery systems, they have also attracted much attention for developing innovative tools for the treatment of bone diseases, such as osteoporosis, non-union bone defects, myelomatosis and bone cancer [209,210,211]. As shown in Figure 7, the large surface-area-to-volume ratio and hollow structure are conducive to enlarging drug-loading capacity, regulating the drug release profile, and enhancing drug permeability and retention. Simple physical adsorption (non-covalent π stacking, hydrogen bonding, and electrostatic interaction) and hydrophobic nature can assist high drug loading of biomolecules like DNA, RNA, and proteins through biological membrane without compromising potency and damaging adjacent cells or tissues. Carbon nanotubes can be easily functionalized by conjugating moieties, such as aptamers, peptides, and small molecules, making them suitable for accumulating in diseased bone tissue, targeting the pathological site, and efficiently delivering specific therapeutic agents [212,213,214]. Furthermore, CNT bioconjugates enhanced the ability of bone nucleus/membrane transport, enabling intracellular targeting delivery to specific subcellular regions [213,215]. Some reports on the advanced applications of CNT-based composites as nanocarrier in bone tissue regeneration and engineering are summarized in Table 2.

### 5.1. CNTs as Nanocarriers for Osteogenic Drugs

It is well known that bone tissue repair and regeneration sometimes require the help of osteogenic drugs or macromolecular proteins. In recent years, some studies have shown that functionalized CNT-based nanomaterials loaded with growth factors, anti-osteoporosis drugs or anti-inflammatory drugs have opened up a brand new way to promote bone tissue repair or treat infections [216]. Dexamethasone (DEX) is an osteogenic drug that promotes gene expression of osteoblasts and induces osteogenic differentiation. Murakami et al. [217] indicated that CNTs with the large surface areas have the capacity to adsorb large amounts of DEX by π–π bonding with aromatic moieties, and DEX-loaded CNTs showed sustained release of DEX in phosphate-buffered saline at 37 °C, providing a good basis and feasibility for drug delivery. Yao et al. [218] used CNTs and silk fibroin to modify the nano-HA/polyamide 66 (nHA/PA66) scaffolds by freeze drying and crosslinking with the aim of loading the DEX. The addition of CNTs not only optimized the mechanical and conductive properties of nHA/PA66 scaffold, but also made their average diameters (~500 µm) and porosity (about 62%) meet the requirements for bone tissue engineering applications. This DEX-loaded scaffold demonstrated an osteogenesis-inducing effect on BMSCs, and DEX at a concentration of 1 mg/mL had the strongest effect different on BMSCs. Since a CNT has a large specific surface area, it can achieve a relatively higher drug loading, whether drugs are adsorbed into the tube hole or attached to the surface. It is noteworthy that CNTs can also improve the efficiency and stability of drug encapsulation and have strong tissue permeability to cross various physiological barriers. Chen at el. [219] prepared CS–CNT-based nanoparticles to control the slow release of isoniazid for the treatment of tuberculosis ulcer. In vitro experiments demonstrated that CS–CNT nanoparticles significantly prolonged the release time over 7 days and stabilized the release rate of isoniazid while retaining the biological function of isoniazid. A further animal model of a tuberculous ulcer indicated that CNTs carried isoniazid to the ulcer site and destroyed mycobacterium tuberculosis. These findings showed the ability to reduce the cytotoxicity and inflammatory response of isoniazid. Therefore, these CNT-based nanocomposites loaded with osteogenic or anti-inflammatory drugs provided a new method for the treatment of bone defects and secondary wounds.

**Figure 8:**
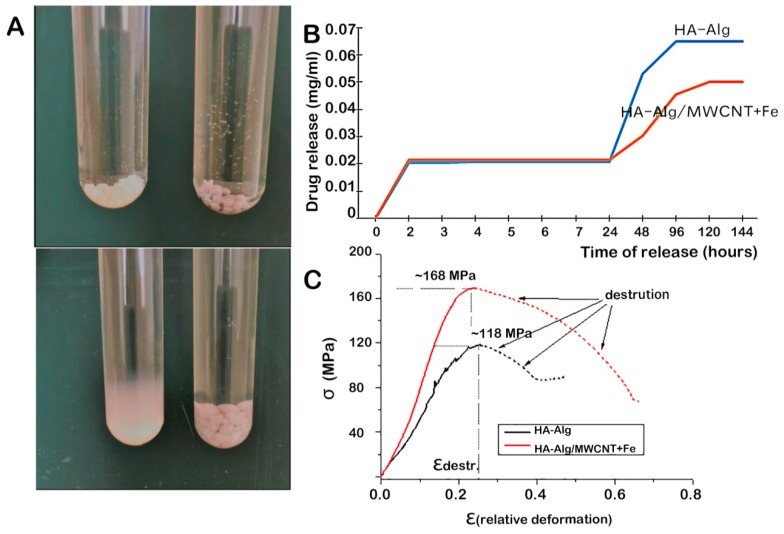
Hydroxyapatite (HA)–alginate (Alg)–multi carbon nanotubes (MWCNT) + Fe beads loaded with chlorhexidine used to fill bone defects. (**A**) The appearances of HA–Alg (left tubes) and HA–Alg–MWCNT + Fe (right tubes) immediately after being placed in PBS on the left figure and after 7 days in a phosphate-buffered saline at 37 °C on the right figure. (**B**) Chlorhexidine release kinetic of the HA–Alg and HA–Alg–MWCNT + Fe beads. (**C**) Compression strength (*σ*) of the HA–Alg and HA–Alg–MWCNT + Fe beads. *ε*_destr_: maximal relative deformations. Reproduced with permission from [221]. Elsevier, 2018.

The controlled release profile of the delivered drugs can directly affect the efficiency of drug action. The preferred CNT as drug carriers are capable of dealing with the limitations of current drug delivery, such as poor drug solubility, rapid inactivation and limited bioavailability. Studies have shown that the presence of CNTs in composite-based materials has good controlled-release properties and prolong the release equilibrium time [220]. Sukhodub et al. [221] fabricated HA–alginate–MWCNT+Fe beads loaded with chlorhexidine which could be used to fill bone defects of a variety of geometries. The presence of MWCNT+Fe adsorbed chlorhexidine and significantly prolonged its release time for 120 h (Figure 8A,B). This new polymer–apatite composite showed a high Young’s modulus comparable to that of steel and had the ability to modulate drug release kinetics (Figure 8B,C), indicating its potential for creating bone tissue model regions capable of withstands mechanical loads. For the formulation of bone implants, Lisa et al. [222] also indicated that CNT, CS, and HA could be used to control the release of different model drugs, such as ibuprofen, ibuprofen sodium, and fluorescein isothiocyanate-dextran. The addition of SWCNTs in combination with CS was able to reduce the total release of the small molecules, ibuprofen, and ibuprofen sodium over 48 h; in addition, the release rate of the hydrophilic macromolecule fluorescein isothiocyanate-dextran was slower than that of ibuprofen and ibuprofen sodium. The results indicated that CNTs showed potential to control the release of both low and high molecular weight hydrophilic drugs, representing a useful multimodal drug delivery platform for bone tissue engineering applications.

In recent years, magnetic-based scaffolds have demonstrated advantages for bone tissue engineering applications and as a promising drug delivery system. Magnetic drug delivery systems have a magnetic response, which can reach the target site and release drugs in a controlled way under the guidance of external magnetic field. These magnetic materials can be activated and attract functionalized magnetic drug nanoparticles, which may open up a fascinating alternative for local pharmaceutical and regenerative therapies in regenerative medicine. Lu et al. [223] prepared foam-like CNT–HA composite scaffolds with superparamagnetic behavior. The foam-like structure demonstrated good mechanical property and optimal pore size, with large pores of 1–2 mm and small pores of 20–300 µm, for osteoconduction and bone ingrowth. Surprisingly, these porous CNT–HA scaffolds showed superparamagnetic behavior at room temperature with saturation magnetization of emu g^−1^ which was favorable for the scaffolds to attract and absorb growth factor stem cells or other bioactive molecules in vivo. Further, Pistone et al. [224] synthesized HA–magnetite–MWCNT nanocomposite decorated with magnetite nanoparticles (MWCNT/Fe_3_O_4_) as bone-specific systems for controlled drug delivery. When clodronate, a well-known osteoporosis drug, was loaded into the nanocomposite, the clodronate-doped nanocomposite had a higher inhibitory effect on osteoclast formation than the single drug. The clodronate-doped nanocomposites showed improved biocompatibility features and magnetic properties induced bone biomineralization, inhibited osteoclast activity in vitro and further positively influenced cellular behavior.

Electrical stimulation plays a role in influencing certain types of cellular behavior, such as adhesion, proliferation and differentiation. Thus, it seems to be a smart way to accelerate bone integration and ingrowth by using electrical stimulation. Vila et al. [225] proposed that external electrical stimulus on conductive CNT–mesoporous-silica composites increased the proliferation of osteoblasts during drug delivery. Carbon nanotubes were uniformly distributed throughout the composite substrates, which ensured the 3D conductive network could be used to transmit the electrical stimuli and affect osteoblasts cultured over the surface. With the application of electrical stimulus on conductive CNT–mesoporous-silica composites, mitochondrial activity increased up to seven times, and osteoblast activity significantly increased with no signs of cell damage, indicating that bone cell metabolism was stimulated. For in vitro drug release experiments with zoledronic acid (Zol) as the model drug, the addition of CNTs increased the drug loading due to the hydrophobic interaction between the CNTs and the Zol molecules, resulting in the retention of more drugs per mg in aqueous media; the increase in conductivity also improved the load capacity of the Zol dosage. Electrophysiological CNTs were able to act as nano-servomotors to store drugs in their cavities during the release experiment thus promoting drug absorption and prolonging drug action time. The possibility of drug delivery in the same process also enhances the potential for local treatment of bone regeneration.

### 5.2. CNTs as Nanocarriers for Proteins, Peptides, and Genes

**Figure 9:**
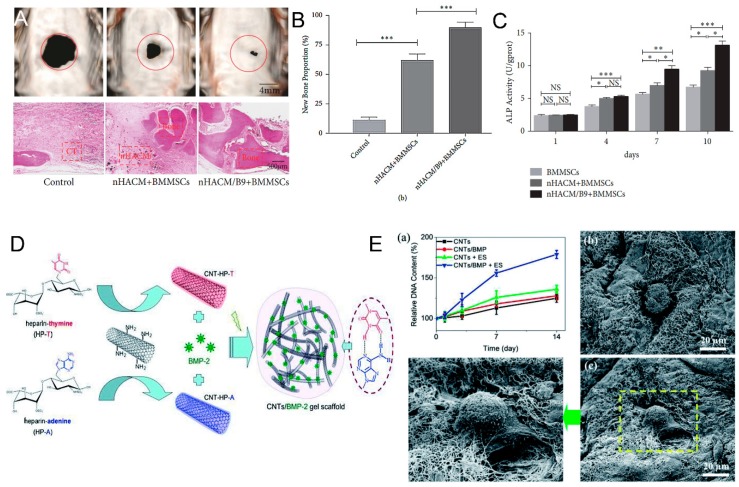
Carbon nanotube (CNT)-based composites as carriers for loading bone morphogenetic proteins (BMPs). (**A**) In vivo radiographic examinations at 12 weeks postoperatively: analysis of the CT images (top) and hematoxylin and eosin staining on the sectioned slices (bottom) showed a significantly larger area of new bone regeneration on the nHACM/B9+BMMSC (nano-hydroxyapatite–collagen I-MWCNT/human bone morphogenetic protein-9 + bone marrow mesenchymal stem cells) groups. (**B**) The proportion of new bone formation in vivo and (**C**) ALP activity in vitro after 4 days of culture in the control, nHACM + BMMSC, and nHACM/B9 + BMMSC groups, indicating that nHACM-B9 scaffolds promoted osteogenic differentiation of BMMSCs. (* *p* < 0.05, ** *p* < 0.01, and *** *p* < 0.001). (**D**) Schematic assembly diagram of a BMP-2-loaded CNT gelatin (gel)-based scaffold via Watson–Crick base pairing. (**E**) Proliferation of human adipose-derived stem cells (ASCs) in a BMP-2-loaded CNT gel-based scaffold under electrically stimulus. (**a**) DNA contents of encapsulated ASCs in CNT gel-based scaffolds as a function of culture time. The DNA content of embedded ASCs in the scaffold changed with culture time. (**b**,**c**) SEM images show the CNT matrices contained in the ASCs and BMP-2 after 7 and 14 days of culture. Reproduced with permission from [226,229]. Hindawi and Royal Society of Chemistry: Open Access, 2019.

Carbon nanotube are unique in that their bioconjugates can simply penetrate the cell membrane and efficiently transport multiple bioactive molecules through endocytosis without interfering with the membrane [225]. Functionalized CNTs can not only be used to deliver multifarious molecular osteogenic drugs, but also has been proved successful nanocarriers of protein, peptides and genes in bone tissue engineering. Bone morphogenetic proteins as specific growth factors are the most effective osteoinductive proteins for bone regeneration. Zhang et al. [226] fabricated a HA–collagen I-MWCNT composite scaffold as a carrier for loading recombinant bone morphogenetic protein-9 (rhBMP-9) to accelerate bone regeneration of critical size defects. The experimental results showed that this novel rhBMP-9-releasing composite scaffold effectively enhanced osteogenic differentiation of bone marrow mesenchymal stem cells (BMMSCs) and induced more new bone formation both in vitro and in vivo (Figure 9A–C), and the proportion of 1.0 wt.% MWCNT was the most suitable for bone tissue engineering. The nanostructured CNT-based materials can increase protein adsorption through stronger interactions, such as van der Waals and electrostatic forces [227]. When the entire superhydrophilic CNT surface was wetted by the protein solution, the contact area between the protein and the nanotube increases significantly, enhancing the interaction and maximizing the retention rate of proteins. In a similar study, Han et al. [228] studied the release spectra of rhBMP-2 bound to two CNT arrays with extreme superhydrophobicity and superhydrophilicity surface. Compared with silicon wafers, the released total amount of rhBMP-2 was significantly reduced on both the superhydrophilic and superhydrophobic CNT arrays. Notably, constant amounts of release were observed on the superhydrophilic CNT arrays at all time intervals, and its cumulative release of 1.87 ± 0.35% at 24 h was a fifth of that of the silicon wafer of 10.04 ± 1.45%. In addition, adding poloxam to the rhBMP-2 load inhibited the large initial burst and further increased the protein release significantly. In a similar study, Qian et al. [229] used CNTs as electrophysiological building blocks for specifically targeted BMP-2 delivery and human adipose-derived stem cells (ASCs) osteogenesis. The CNT gel-based scaffold utilized Watson–Crick base pairing mechanism to bond with adenine- and thymine-functionalized heparin derivatives which were able to stabilize BMP-2 activity, bind cell growth factors, and prolong delivery time through affinity (Figure 9D). The BMP-2-loaded CNT gel-based scaffold exhibited the prominent mechanical integrity and advanced electro-physiological functions; compared with ASCs cultured on pristine hydrogels, their spontaneous osteogenesis on bioelectrical gel scaffolds increased by approximately 400%. Specifically, the electro-conductibility network of this CNT scaffold encapsulated with nanofibrous architectures was a key feature of the cell scaffolds, which enhanced the differentiation and organization of ASCs by adding extra electrical stimulus (Figure 9E). These findings indicated that CNT-based scaffolds loaded with different types of BMPs have the potential to possess a controlled and sustainable release profile and promote osteoblast differentiation and bone formation. Further, it is notable that CNT-based electrophysiological materials may provide a new concept for inducing spontaneous osteogenesis and accelerating the healing of bone defects.

**Figure 10:**
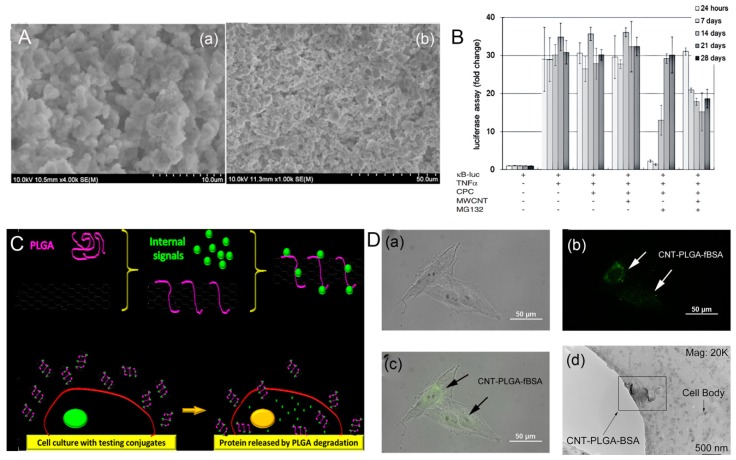
Carbon nanotube (CNT)-based delivery systems for peptides and proteases. (**A**) SEM images of calcium phosphate cement (CPC)–MWCNT–Z-Leu-Leu-Leu-al (MG132). (**B**) The luciferase assay shows that MG132 released from CPC or CPC–MWCNT inhibited TNF-α-induced NF-κB activation and osteoclast differentiation. (**C**) Schematic diagram of CNT–poly(lactide-co-glycolide) (PLGA) conjugate fabrication and cell-to-cell delivery. (**D**) Penetration of CNT–PLGA–proteins into osteosarcoma cells cultured in CNT–PLGA–fBSA (fluorescent bovine serum albumin) (**a**,**b**) and CNT–PLGA–BSA penetrating the osteosarcoma cell (**c**,**d**). Reproduced with permission from [230,233]. Elsevier, 2014; PLOS: Open Access, 2013.

As for the peptides and small compounds, Z-Leu-Leu-Leu-al (MG132), as a potent proteasome inhibitor, has the potential to reduce the absorption of osteoclasts. However, since MG132 can induce apoptosis, its sudden release may cause damage to surrounding cells and tissues. Lin et al. [230] investigated the release pattern of carboxylic acid-functionalized MWCNT-reinforced monetite-based CPCs as drug carriers for MG132 (Figure 10A). Surprisingly, the cell viability of MC3T3-E1 cells did not significantly decrease when MWCNT was present in cement, compared with the mass death of osteoblasts MC3T3-E1 cells cultured in the MG132-loaded CPC medium. Z-Leu-Leu-Leu-al contains three leucine amino acids and one aromatic ring that make it highly absorbable on the surface of CNTs via non-covalent π-stacking or hydrophobic interactions [231]. Although both CPC and MWCNT-reinforced CPC were able to carry MG132 and remained effective in inhibiting cytokine-induced NF-κB activation, the addition of MWCNTs to CPCs retarded the release rate of MG132 within 24 h and attenuated its burst release. This suggested a possible solution for improving the drug delivery property of CPCs (Figure 10B). The results of this study showed that CNTs have the ability to deliver bioactive peptides and could regulate the burst release of peptides in the delivery system, thus enhancing their biological functions in bone tissue repair.

Functionalized CNT-based systems have been rigorously investigated as potential successful pathways for gene therapy, especially cancer therapy. For instance, Anderson et al. [165] successfully prepared functionalized SWCNTs as efficient siRNA carriers to deliver siRNA into cancer cells, suppressing the growth of the cancer cells. Geyik et al. [232] indicated that the covalent bioconjugate of MWCNTs with cationic groups enabled amino-modified linearized plasmid DNA to be delivered into cells. However, specific gene-loaded CNT delivery systems for bone tumors and other bone diseases have not been widely reported. Cheng et al. [233] successfully engineered a gene, transcription factor, and signal molecular delivery system based on PLGA-functionalized CNTs for bone tissue engineering. The carboxylated CNTs coated with PLGA were functionalized with pro-apoptotic protein caspase-3 (CP3) to induce cell apoptosis. These CNT–PLGA conjugates were able to penetrate into MG-63 osteosarcoma cells and release CP3 through the degradation of PLGA, as depicted in Figure 10C. The CNT–PLGA–fBSA (fluorescent bovine serum albumin) conjugates promoted the transmission of RNA and transcription factors to cells and showed a pronounced cell penetration ability (Figure 10D), suggesting that CNT could deliver substances across membranes. These CNT–PLGA conjugates not only had a reliable time-dependent drug release profile, but also a high transfection rate, which was 30% or 40% higher than that of polymers and liposome nanoparticles. Therefore, CNT-based nanocarriers show an efficient and promising application in non-viral gene delivery systems, opening up a novel avenue for bone tumors and other bone diseases.

## 6. Conclusions and Future Prospects

The biomedical applications of CNT nanomaterials have been one of the most productive sub-areas of nanomedicine and nanobiotechnology. Studies on CNT-based biomaterials and their derivatives favor an incredible combination of physical and biological sciences for enhancing cellular interactions, adhesion, proliferation, and osteogenesis differentiation. Extensive research is under way to combine CNTs with existing biomaterials to develop new and highly functional tissue-engineered implants and scaffolds for bone repair and regeneration. These CNT-based or -reinforced scaffolds have a promising biocompatibility and biophysicochemical properties which can promote osteoblast differentiation and guide bone regeneration in bone tissue engineering. Moreover, the CNTs with large surface area and good biocompatibility and stimulation have been extended in their scope as nanocarriers for various drug delivery and cellular transport systems in the treatment of bone defects and diseases. Carbon nanotube-based biomaterials have shown great potential in the delivery of different therapeutic agents, such as drugs, peptides, proteins, and genes, since their large surface and hollow structure can adsorb the osteogenic drug, macromolecular, and protein via π–π stacking, hydrogen bonding, and electrostatic interaction, with high loading and good efficiency. Furthermore, CNTs can cross the bone cell nucleus/membranes and transport intracellular-targeted drugs to specific subcellular regions in a controlled manner, which enhance their role as preferred nanocarriers. Considering the above merits, CNT-based biomaterials offer tremendous potential to be used as novel biomaterials in bone tissue engineering.

Despite these substantial advances and progress in the application of CNTs for bone regeneration and repair, there are still a number of difficulties from experimental research to clinical application which need to be overcome by the joint efforts of scholars and researchers all over the world. Firstly, follow-up studies on the low cytotoxicity and bioavailability of CNT-based biomaterials will require a comprehensive toxicological analysis to ensure their safe clinical applications. Biofunctionalized CNT-based composites and substrates have been shown to be biosafe and biocompatible for use as local bone scaffolds or implants, but should be avoided from loosening and migrating into the bloodstream, lung, and abdominal cavity. More attention should be paid to the in vitro and in vivo biosafety studies of CNT-based biomaterials for further understanding of the complex interactions between cells and materials. The functionalization of CNTs with covalent and non-covalent schemes is the most basic and effective method to reduce biotoxicity, enhance solubility, and combine various biological molecules; therefore, novel and creative technologies and strategies need to be further studied and developed to modify these tubular structures. Secondly, it is noteworthy that CNTs have outstanding capabilities in promoting and inducing stem cell differentiation into specific lineages, especially osteogenic differentiation. This could be attributed to the surface interaction between the cell membrane and the CNT-based biomaterial which has a positive effect on cell behavior through absorption or repulsion of specific differentiation factors. It requires a deeper understanding of the underlying physiological mechanisms of CNT–cell (or tissue and organ) interactions, as well as their behaviors in complex microenvironments and their long-term biocompatibility. Thirdly, in terms of mechanical properties, CNT-based biomaterials have been considered as the most suitable scaffold materials to overcome mechanical strength differences in bone tissue engineering for many years, due to the fact of their large surface area, high porosity, and effective biological interaction. However, biomechanical considerations warrant further studies to verify the thresholds of mechanical stimuli of CNTs as bone tissue engineered scaffolds or implants, ensuring that the process of bone remodeling can be initiated and restored. Next, the unique electrical properties of CNTs provide a valuable opportunity to be used as biomaterials combined with efficient electrical stimulus to stimulate certain cellular behavior. This smart approach should also receive due attention to accelerate bone integration and growth through using certain electrical stimulation. Finally, various novel CNT-based delivery systems should be further designed, developed, and evaluated in vitro and in vivo to fabricate innovative tools for enhancing osteogenesis, accelerating bone tissue repair, as well as directly treating bone diseases, such as osteoporosis, non-union bone defects, myelomatosis, and bone cancer. Overall, CNTs are emerging as superior materials capable of providing innovative strategies and future prospects for bone tissue regeneration and engineering. However, many challenges remain to be overcome in order to achieve the ultimate aspiration of the transition from experimental research to clinical practice.

## Figures and Tables

**Table 1:** Applications of CNT-based nanomaterials as scaffolds or implants in bone tissue.

Substrate Materials	CNT Application	Consequences	References
	**Calcium Phosphate**		
hydroxyapatite (HA)	bone implant materials	➢ enhanced mechanical properties ➢ increased proliferation of fibroblasts and osteoblast ➢ enhanced bone integration in vivo	[147,148]
hydroxyapatite (HA)	coating material for implants	➢ promoted apatite mineralization ➢ accelerated new bone formation	[149]
beta-tricalcium phosphate (β-TCP)	bone repair biomaterials	➢ induced apatite formation ➢ enhanced HA formation	[154]
calcium phosphate cements (CPC)	injectable bone substitutes	➢ increased compressive strengths ➢ promoted the nucleation, growth, and formation of HA crystals	[157]
	**Natural Polymers**		
chitosan (CS)	nanocomposite films	➢ improved elastic modulus and tensile strength ➢ improved bioactive properties	[62]
chitosan (CS)	bone tissue scaffolds	➢ increased water uptake ability and porosity ➢ enhanced cell proliferation, protein content, alkaline phosphatase, and mineralization ➢ promote the ectopic bone formation	[159,163]
chitosan(CS)–hydroxyapatite (HA)	bone tissue engineering	➢ increased elastic modulus and compressive strength ➢ adsorbed and released protein ➢ enhanced cell proliferation osteoconduction and bone generation	[162,163]
silver sulfadiazine (AgSD)–chitosan (CS) nanofiber	coating material for implants	➢ exhibited exceptional antibacterial performance ➢ enhanced cellular compatibility proliferation ➢ reduced the incidence of bone infections	[94]
collagen	bone repair biomaterials	➢ improved mechanical stability ➢ enhance the construct functionality	[172]
collagen	3D CNT-coated bone scaffolds	➢ accelerated early differentiation of osteoblasts ➢ induced new bone formation in vivo	[175]
collagen–hydroxyapatite (HA)	bone tissue scaffolds	➢ increased the stiffer ➢ promoted bone marrow mesenchymal stem proliferation and spreading ➢ promoted mRNA and protein expressions of bone sialoprotein and osteocalcin	[150]
gelatin–hydroxyapatite (HA)	artificial bone grafts	➢ exhibited similar structure and composition to natural bone ➢ increased elastic modulus, tensile strength, and elongation rate ➢ increased cell viability	[176]
gelatin–chitosan (CS)	bone scaffold materials	➢ improved mechanical strength and elastic modulus ➢ enhanced MC3T3-E1 cell adhesion, proliferation and osteogenesis differentiation	[177]
bacterial cellulose	bone tissue scaffolds	➢ increased mechanical properties ➢ supported osteoblast viability, adhesion and proliferation	[179]
silk fibroin	nanocomposite films	➢ supported bone cell adhesion and growth	[180]
	**Synthetic Polymers**		
polycaprolactone (PCL)	3D bone scaffolds	➢ Increased tensile and compressive strength ➢ improved cell attachment proliferation and differentiation, and protein adsorption	[187,189]
polycaprolactone (PCL)–hydroxyapatite (HA)	3D bone scaffolds	➢ improved compressive strength and elastic modulus ➢ induced substantial mineralization of apatite ➢ supported cellular growth, angiogenesis, and tissue development	[190]
polymethyl-methacrylate (PMMA)	bone cements	➢ improved the fatigue properties ➢ permitted the cell adhesion and growth	[191]
polymethyl-methacrylate (PMMA)	bone cements	➢ promoted cell adhesion ➢ induced osteogenic differentiation ➢ promoted osseointegration	[192]
Poly(lactide-co-glycolide) (PLGA)	load-bearing bone tissue scaffolds	➢ improved the polymeric scaffold’s mechanical strength ➢ displayed good cellular and tissue compatibility,	[194]
Poly(lactide-co-glycolide) (PLGA)	bone repair scaffolds	➢ enhanced the mechanical strength ➢ controllable surface roughness ➢ increased osteoblasts attachment and proliferation	[195,196]
polylactic acid (PLA)	nanocomposite materials	➢ increased the tensile strength, elongation at break and impact strength ➢ obtained a higher thermal stability	[197]
polylactic acid (PLA)	bone tissue engineering	➢ exhibited electrical conductivity ➢ promoted elongation and outgrowth of osteoblasts by electrical stimulation	[199]
poly-L-lactic acid (PLLA)	bone tissue engineering	➢ improved Young’s modulus ➢ supported the adhesion and proliferation of human bone marrow stromal cells (BMSCs)	[198]
polyvinyl alcohol (PVA)–chitosan (CS)	bone tissue engineering	➢ exhibited small diameters (~160 nm) and high porosity ➢ adsorbed much more protein ➢ improved the cell response and proliferation	[207]
poly(etheretherketone)-calcium polyphosphate cements (CPPs)	load-bearing orthopedic application	➢ enhanced mechanical performance close to or higher than human cortical bone ➢ promoted initial cell adhesion, viability and osteogenic differentiation	[208]

**Table 2:** Applications of CNT composites as nanocarriers for bone tissue regeneration and engineering.

Delivery System	Drugs/Molecular Type	Consequences	References
carbon nanotube (CNTs)/silk fibroin–hydroxyapatite (HA)/polyamide 66 (nHA/PA66) scaffolds	Dexamethasone (DEX)	Promoted the expression of osteoblast genes and induced the osteogenic differentiation	[217,218]
Chitosan (CS)–CNTs nanoparticles	isoniazid	Prolonged the release time, stabilized the release rate of isoniazid, retained the biological function, and reduced the cytotoxicity and inflammatory response of isoniazid	[219]
HA–alginate–MWCNT + Fe beads	chlorhexidine	Prolonged chlorhexidine release time and showed high a young’s modulus comparable to steel	[221]
CNT–chitosan (CS)–hydroxyapatite (HA) composite materials	ibuprofen (IBU) ibuprofen sodium (IBU-Na) fluorescein isothiocyanate-dextran (FITC-Dex)	Controlled the release of both low and high molecular weight hydrophilic drugs	[222]
HA–magnetite–MWCNT nanocomposite with magnetite nanoparticles (MWCNT/Fe_3_O_4_)	clodronate	Improved magnetic properties, induced bone biomineralization, and inhibited osteoclast activity in vitro	[223,224]
CNT–mesoporous silica composites	zoledronic acid (Zol)	Ensured the 3D conductive network to transmit the electrical stimuli, affected osteoblasts cultured over the surface, and increased the drug loading	[225]
CNT gel scaffold via specific pairing of functionalized nucleobases	human bone morphogenetic protein-2 (BMP-2)	Significantly increased the spontaneous osteogenesis on bio-electrical gel scaffolds and enhanced cell differentiation and organization via extra electrical stimulus.	[229]
CNT arrays	recombinant human bone morphogenetic protein-2 (rhBMP-2), poloxamer	Retained a larger amount of rhBMP-2, delayed protein release and inhibited the large initial burst	[228]
hydroxyapatite (HA)–collagen–MWCNT composite scaffolds	recombinant bone morphogenetic protein-9 (BMP-9)	Enhanced osteogenic differentiation in vitro and induced more new bone formation in vivo	[226]
carboxylic acid-functionalized MWCNT–monetite-based CPC	Z-Leu-Leu-Leu-al (MG132)	Exhibited a sustained drug release, and confirmed the therapeutic effect by the inhibition of cytokine-induced osteoclast differentiation	[222]
poly(lactic-co-glycolic) (PLGA)-functionalized CNTs materials	pro-apoptotic protein caspase-3 (CP3)	Promoted delivery of RNA and transcription factor to cells and demonstrated a pronounced ability of cell penetration	[233]

## Abbreviations

CNT	carbon nanotube
HA	hydroxyapatite
ECM	extracellular matrix
ALP	alkaline phosphatase
TCP	tricalcium phosphate
β-TCP	Beta-tricalcium phosphate
CPC	calcium phosphate cement
BMP	bone morphogenetic protein
rhBMP-2	recombinant human bone morphogenetic protein-2
rhBMP-9	recombinant human bone morphogenetic protein-9
ASC	human adipose-derived stem cell
MSC	mesenchymal stem cell
BMSC	bone marrow stem cell
BSA	bovine serum albumin
PCL	polycaprolactone
PMMA	polymethyl methacrylate
PLGA	poly(lactide-co-glycolide)
PLA	polylactic acid
PLLA	poly-L-lactic acid
PVA	polyvinyl alcohol
PGA	poly glycolic acid
PEEK	poly(etheretherketone)
PPF	polyanhydrides poly(propylene fumarate)
DEX	dexamethasone
Zol	zoledronic acid
MG132	Z-Leu-Leu-Leu-al
CP3	pro-apoptotic protein caspase-3
